# Emerging Therapies for Neurological Disorders: A Clinical Review of MANAGED (Music, Art, Nature-Based, Animal-Assisted, Game, Essential Oil, Dance) Care

**DOI:** 10.3390/neurosci6020051

**Published:** 2025-06-04

**Authors:** Alyssa Wan-Chei Lee, Rahim Hirani, Jonathan Ogulnick, Raj K. Tiwari, Mill Etienne

**Affiliations:** 1 School of Medicine, New York Medical College, Valhalla 10595 NY, USArhirani2@student.nymc.edu (R.H.);; 2 Graduate School of Biomedical Sciences, New York Medical College, Valhalla 10595 NY, USA; 3 Department of Neurology, Renaissance School of Medicine, Stony Brook University, Stony Brook 11794 NY, USA; 4 Department of Neurology, New York Medical College, Valhalla 10595 NY, USA

**Keywords:** neurological disorders, alternative therapies, holistic approach, quality of life, healthcare costs, clinical practice

## Abstract

In the face of the limitations in pharmacological and surgical interventions for neurological conditions such as Parkinson’s and Alzheimer’s disease, patients are increasingly turning to non-pharmacological and alternative therapies to manage their symptoms and improve their quality of life. This shift underscores the urgent need for accessible, effective, and affordable treatments. This literature review examines a range of alternative and personalized therapies, including game therapy, animal-assisted therapy, dance therapy, art therapy, music therapy, aroma therapy, and shinrin-yoku therapy. These modalities have demonstrated promising results in mitigating symptoms and enhancing well-being among individuals grappling with neurological disorders. Moreover, these therapies offer a holistic approach that complements traditional medical interventions, underscoring the importance of integrating diverse treatment modalities. Despite their historical roots in non-clinical settings, their potential in modern clinical practice remains untapped. The findings suggest the necessity for further research, particularly large cohort studies, to validate the efficacy of these personalized therapies and advocate for their widespread adoption. In an era marked by escalating healthcare costs, the exploration of alternative therapies presents a compelling avenue for enhancing patient care while simultaneously addressing economic challenges within the healthcare system.

## 1. Introduction

Alternative therapies and medicine have a long-standing history. What we currently refer to as alternative medicine was, in its time, regarded as conventional medicine by those living in that era. For instance, there is evidence that acupuncture was being used by Chinese Emperor Huangdi as early as 2500 BC. Some evidence points to its utilization starting as early as the Neolithic era, from 5000–6000 BC [1]. More recent studies have found that acupuncture may be useful in the treatment of neurodegenerative diseases such as Alzheimer’s disease (AD), Parkinson’s disease (PD), and amyotrophic lateral sclerosis (ALS) via inhibition of the accumulation of toxic proteins such as amyloid-β (hallmark of AD), metabolic activity modulation, and depression of neuronal apoptosis [2]. Acupuncture appears to have a neuroprotective effect in both humans and animal models of disease, and increased activity in areas of the cerebral cortex associated with cognition can also be seen on fMRI after using acupuncture [3]. Ayurveda, translated from Sanskrit as “The Science of Life”, is another form of alternative medicine rooted in Ancient India. Its healing tenets primarily call for maintaining a balance of the three doshas, or energies, in the body. The methodologies of treatment include panchakarma (“cleansing” or purging of the body), oil treatments, and ingestion of herbal concoctions. Similarly to acupuncture, Ayurveda appears to have a profound impact on managing and treating neurological issues. Severe neurological and motor neuron deficits following spinal cord injury have been shown to greatly improve with the use of oil and ghrita Ayurveda therapies [4]. Alternative therapies have been used extensively for centuries if not millennia and there are also historical and scientific precedents for their utility and promise in modern clinical neurology.

The current standard of care for most neurological conditions includes pharmaceuticals and other procedures that for some patients are cost-prohibitive, may have limited efficacy and may have intolerable side effects/toxicities [5,6,7]. Surgical procedures are often expensive, overly invasive, and not consistently curative. For instance, even the most advanced surgical techniques in the treatment of Parkinson’s disease such as deep brain stimulation (DBS), magnetogenetics, and sonogenetics have been demonstrated to be generally limited to easing the severity of motor symptoms such as bradykinesia, rigidity, tremor and medication-induced dyskinesias [8]. Cholinesterase inhibitors such as donepezil, rivastigmine, and galantamine can be used to treat some of the cognitive symptoms of Alzheimer’s disease; however, these drugs have only been shown to provide symptomatic relief in patients without altering the original disease course. Additionally, these pharmaceutical interventions often result in deleterious side effects; for example, rivastigmine can cause bradycardia, seizures, and Stevens–Johnson syndrome in predisposed individuals [9].

Neurologists and neurosurgeons should consider complementary treatment modalities for patients who either present with financial barriers to accessing traditional care or have conditions that are refractory to the typically indicated treatments. There is also an argument to be made for starting some form of alternative therapy as soon as a patient is diagnosed, given their numerous clinical benefits and often favorable side effect profiles. Alternative therapy regimens can also be tailored to each patient’s needs, interests, and symptoms. Since many, if not all, are non-invasive, there is a lower risk for adverse side effects or toxicities. Patients may also be more willing and able to perform an activity such as coloring by number or playing a game as opposed to consistently taking a pill multiple times per day increasing the probability that the patient will adhere to their treatment regimen [10]. There is evidence to suggest that traditional medicine may be limited in terms of its efficacy in treating certain neurological conditions, especially those related to the pathogenesis of neuropathic pain; in contrast, alternative therapies including nutrition, exercise, mind–body medicine, supplements, and acupuncture have been demonstrated to provide relief to these patients. This is especially significant in conditions for which we have no definitive cure, including carpel tunnel syndrome, migraines, and dementia [11].

This comprehensive review explores seven emerging alternative treatment modalities in neurology, supplemented with evidence-based reasoning to highlight their potential benefits for patients with diverse neurological conditions. These therapies include Music therapy, Art therapy, Nature-based therapy, Animal-assisted therapy, Game-based therapy, Essential oil therapy, and Dance therapy, collectively remembered through the mnemonic MANAGED care. It is important to emphasize that while these therapies show promise in recent studies and are gaining traction in the field, they are not exhaustive; many other alternative therapies may also offer substantial benefits.

Databases searched include PubMed, Google Scholar, and ScienceDirect. Search terms used include “art therapy”, “music therapy”, “nature therapy”, “shinrin-yoku”, “animal assisted therapy”, “animal therapy”, “game therapy”, “aromatherapy”, “essential oils”, “dance therapy”, and “dance movement therapy”. The literature review timeframe that was utilized was from 1990–2024. We established clear inclusion and exclusion criteria to ensure a focused and rigorous analysis of relevant studies. We included clinical trials, observational studies, systematic reviews, meta-analyses, and case studies that specifically investigate the effectiveness of MANAGED therapies (Music, Art, Nature-Based, Animal-Assisted, Game, Essential Oil, Dance) in treating neurological disorders such as Alzheimer’s disease, Parkinson’s disease, multiple sclerosis, epilepsy, stroke, traumatic brain injury, anxiety, and depression. Only studies that directly examined these therapies as interventions and measure their impact on cognitive, emotional, physical, or psychological outcomes were considered. Studies must have involved patients diagnosed with neurological disorders across various stages and ages, and must have been published in English or with available English translations.

We excluded non-clinical studies such as theoretical papers, opinion pieces, commentaries, and editorials that did not provide empirical data. Studies focusing on therapies unrelated to the MANAGED acronym, including pharmacological or surgical treatments, were also excluded. Studies addressing non-neurological disorders or those without adequate data (e.g., small sample sizes or poor methodological quality) were excluded from the review. Additionally, non-English studies were excluded unless a translation was available. We also excluded abstracts, conference proceedings, and unpublished works unless full-text versions were available for review. These criteria ensure that our review is comprehensive, focused, and based on high-quality evidence regarding the effectiveness of emerging therapies for neurological disorders.

## 2. Music Therapy

Music therapy is one of the most extensively studied forms of alternative therapy, with evidence of its use present in ancient civilizations including Mesopotamia, Egypt, Greek Antiquity, the Middle Ages, the Renaissance, and Baroque periods [12]. Both listening and producing music via instruments and/or voice have been associated with positive effects in neurological problems. It has been tied to improvements in a variety of neurological conditions, including coma, traumatic brain injury (TBI), dementia, epilepsy, and stroke. According to a systematic review by Zaatar et al., music therapy engages a broad and interconnected network of specific brain regions involved in sensory, cognitive, emotional, and motor functions. The auditory cortex, particularly the superior temporal gyrus, is activated during music listening as it processes sound. The prefrontal cortex plays a key role in executive functions such as attention, planning, and working memory, all of which are stimulated by music. The hippocampus, essential for memory formation and retrieval, is notably influenced by music, especially in evoking autobiographical memories—an effect beneficial in conditions like dementia and Alzheimer’s disease. Emotional responses to music involve the amygdala, while the nucleus accumbens, part of the brain’s reward circuitry, is activated during pleasurable music experiences, leading to dopamine release and a sense of enjoyment.

Music also stimulates the motor cortex and cerebellum, both crucial for movement coordination and rhythm processing, which is particularly relevant in motor rehabilitation settings such as stroke recovery. Furthermore, the default mode network (DMN)—which includes areas like the medial prefrontal cortex, posterior cingulate cortex, and angular gyrus—is synchronized during music listening, supporting introspection, creativity, and emotional regulation. The insula, involved in emotional awareness and empathy, is also active during emotionally resonant music experiences. Altogether, these findings highlight how music therapy taps into a wide array of brain systems, making it a powerful tool for enhancing cognitive, emotional, and motor functions [13].

Sun et al. demonstrated that after being exposed to music therapy, patients in TBI-induced coma showed significant improvement in both Glasgow Coma Score (GCS) and quantitative EEG value [14]. In dementia patients, a meta-analysis showed that music therapy produced significant improvements in disruptive behaviors, anxiety, depression, and cognitive functioning [15]. A review by Sihovenen et al. demonstrated benefits of music therapy in rehabilitating patients suffering from a diverse range of neurological diseases such as stroke, dementia, multiple sclerosis, and epilepsy [16]. Another study evaluated the impact of music on rehabilitation by compiling studies demonstrating its effect on neuronal plasticity. It does so by increasing cerebral blood flow to bilateral parts of the brain, particularly through the middle cerebral artery (MCA). Increased blood flow through the MCA has the potential to augment neuronal plasticity by supplying oxygen and other nutrients to specific areas of the brain. This effect is especially potent in patients living with the residual effects and long-lasting sequelae of MCA infarction [17]. Additionally, the authors explained the role of music in increasing the activity of the dopaminergic mesolimbic system. This increased activity is likely responsible for the mood improvement in patients treated with music therapy. Of note, melodic intonation therapy was utilized by Congresswoman Gabrielle Giffords who suffered from extensive severe traumatic brain injury with aphasia from a gunshot wound to the head after a mass shooting in Arizona. Furthermore, Liao et al. compiled numerous studies showing that for patients with refractory epilepsy, music therapy can help reduce the frequency of epileptiform discharges. The mechanism by which this effect is put in place is not entirely clear; however, researchers hypothesize that resonance, mirror neurons, dopamine pathways, and parasympathetic activation may be involved [18].

Music therapy can have numerous benefits in patients with movement disorders such as Parkinson’s disease and Huntington’s disease. Numerous studies have reported short-term benefits of rhythmic auditory stimulation on gait deficiencies, including gait freezing in PD. Music therapy may therefore be used to reduce fall occurrence in PD patients [19]. Improvements in gait in both the dopaminergic “on” and “off” states implies that music therapy may be a useful and even integral tool in the clinical arsenal of PD therapies, particularly in the treatment of gait abnormalities [20,21]. PD patients who engage in group singing, dancing, and playing instruments have demonstrated remarkable improvement in both motor and non-motor PD symptoms [22]. In particular, singing therapy in group and individual settings has been shown to significantly improve communication ability, emotional regulation, breathing, and swallowing motor control in patients with PD [23]. In Huntington’s disease patient tools such as the Music Therapy Assessment Tool for Advanced Huntington’s disease (MATA-HD) can be utilized to quantify degree of improvement with the application of music therapy [24]. In addition to PD, movement disorders such as Tourette syndrome and progressive supranuclear palsy show encouraging evidence for the effectiveness of music therapy, though more research is needed to solidify and enhance this promising area of treatment [25,26]. Recent studies have also highlighted the neuroplastic effects of music therapy, suggesting that it may not only alleviate symptoms but also promote neural reorganization, particularly in regions related to motor control and emotional regulation [27]. Furthermore, promising findings from small-scale studies on neurodegenerative diseases indicate that music therapy, combined with other therapeutic interventions like physical therapy or cognitive training, may provide even more comprehensive benefits [28,29]. Future research should investigate the mechanisms through which music influences specific pathways and brain regions in neurological disorders, as well as the implementation of multicenter trials to demonstrate the efficacy of music therapy. This line of investigation has the potential to uncover valuable insights and enhance therapeutic approaches in this field.

**Figure 1:**
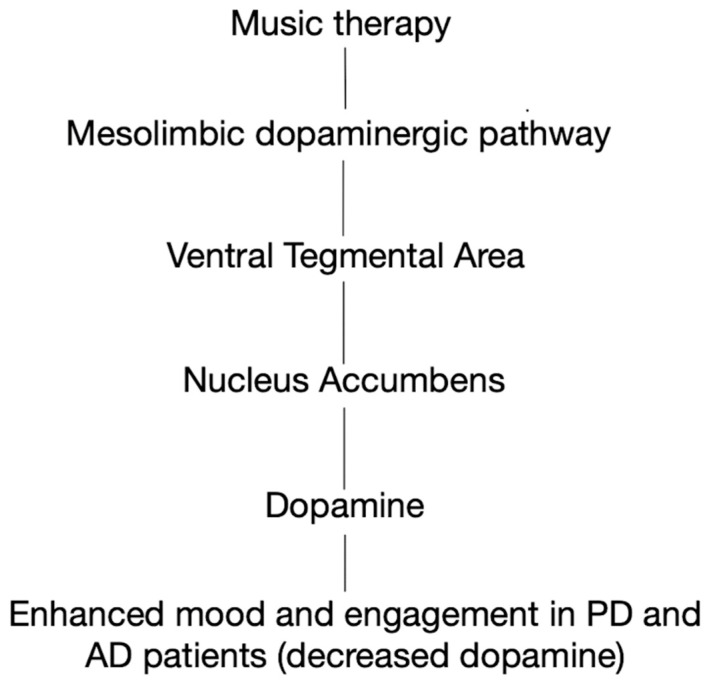
Activation of the brain’s reward system resulting in increased dopamine to treat degenerativeative diseases. Listening to music stimulates the brain’s reward circuitry, particularly the mesolimbic dopaminergic pathway, which includes the ventral tegmental area and the nucleus accumbens. This activation leads to the release of dopamine, a neurotransmitter associated with pleasure and motivation. In neurodegenerative diseases like Alzheimer’s and Parkinson’s, where dopaminergic systems are compromised, music-induced dopamine release may enhance mood and engagement [30].

Audio frequencies, particularly those in the range of 10 Hz to 100 Hz, have been explored for their potential therapeutic effects in neurodegenerative disorders. These low-frequency sounds are thought to influence brain activity, potentially enhancing neuroplasticity and modulating neuronal excitability. For example, 10 Hz frequencies, commonly associated with alpha waves, have been shown to promote relaxation and improve cognitive function, which may be beneficial for conditions such as Alzheimer’s disease and Parkinson’s disease. Frequencies in the range of 40 Hz, often linked with gamma brain waves, have also been studied for their potential to enhance synaptic activity and reduce amyloid plaque formation, a hallmark of Alzheimer’s disease. Moreover, low-frequency sound stimulation (20–50 Hz) may have a neuroprotective effect by promoting mitochondrial health and reducing oxidative stress, both critical factors in the progression of neurodegenerative diseases. However, while the preliminary results are promising, further clinical research is required to fully understand the efficacy and mechanisms behind audio frequency-based therapies for neurodegenerative disorders. Figure 1 depicts how music therapy activates the mesolimbic dopaminergic pathway, engaging the ventral tegmental area and nucleus accumbens to stimulate dopamine release, which may enhance mood and engagement in patients with PD and AD, where dopamine levels are typically reduced.

## 3. Art Therapy

Similarly to MT, art therapy is another highly accessible form of psychological and physiotherapy with extensively documented therapeutic capabilities. Art therapy has healing potential across a wide range of neurologic disabilities due to its multimodal integration of skills including visuospatial processing, abstraction ability, memory, sensorimotor activity, and hand–eye coordination [31].

Art therapy is particularly useful in patients with dementia, stroke, epilepsy, and Alzheimer’s disease [32,33,34]. There is a valuable opportunity to incorporate art therapy in the treatment of patients with dementia, as it can provide meaningful benefits where medications fall short. A study by Windle et al. examined how effective specific types of art therapy are in dementia patients, the respective clinical effects of each type of art therapy, and the mechanisms behind their efficacy. They found that the aesthetic experiences of observing art, and the dynamic creation of art were both effective in treating patients with dementia. An aesthetic experience is defined here as the emotions and therapeutic catharsis associated with creating and visualizing art. Specifically, they found that these forms of art therapy were correlated with improvements in psychological well-being, cognitive processes such as recall, and feelings of social connectedness [35]. In light of the fact that many art forms transcend language barriers, the treatment of migrant populations with traumatic stress disorders and anxiety with art therapy has proven to be effective in reducing the severity of those disorders [36]. In patients with debilitating migraines, art therapy may present an avenue for amelioration of symptoms [37]. Along the same lines, art therapy has been successfully applied to chronic pain management, although these results have been inconsistent and require further study [38]. Art therapy used with inpatient pediatric oncology patients also results in improved mental health and reduces the severity of adverse side effects from treatments such as chemotherapy [39]. In patients with combat-related posttraumatic stress disorder (PTSD), art therapy in combination with Cognitive Processing Therapy (CPT) has been shown to improve trauma processing. Veterans also considered art therapy and CPT to be an integral component of their treatment plan in light of the fact that it provided healthy distancing, enhanced trauma recall, and increased access to emotions [40].

Multiple studies have demonstrated significant improvements in mood and well-being in epileptic patients following administration of art therapy, which may translate to improved quality of life. For instance, Stafstrom et al.’s use of art therapy in their patient population resulted in uplifted mood and high satisfaction in pediatric epilepsy patients and their parents, both of whom demonstrated strong approval of the program [41]. A study of 50 hospitalized patients from Turkey (17 with epilepsy and 33 with stroke) demonstrated a significant reduction in the Beck Hopelessness Scale (BHS) after engaging with clay therapy [42]. Patients with epilepsy who have difficulty adapting to social situations had an increased degree of self-expression and emotional regulation following the use of scratch art therapy [43]. Although art therapy may not directly alleviate seizures in patients with epilepsy, it is a cost-effective approach to enhance mood and outlook on life particularly in group settings, something that is arguably just as integral to human health and well-being as medical treatment [41]. Parkinson’s disease patients can also benefit from the use of art therapy. Improvements in motor and visuospatial processing, cognition, mood, motivation, self-image, self-efficacy, interpersonal functioning, creativity, and overall level of functioning are seen following art therapy [31]. Notably, there is no standard set of therapeutic exercises for art therapy interventions nor are there universally applied neuroaesthetic principles in this modality [44]. In light of this fact, art therapists and other medical professionals may benefit from discussing and setting a standardized set of art therapeutic principles and activities that should be included in the art therapeutic protocol based on existing scientific data on efficacy.

## 4. Shinrin-Yoku (“Forest Bathing”)

Shinrin-yoku, translated directly as “forest bathing” is defined as the therapeutic practice of spending time in a forest or other natural setting to engage with one’s sensory connection to their surroundings. Shinrin-yoku has been reliably tied to both psychological and physiological relaxation. In a field experiment and study conducted across 24 forests in Japan, 12 individuals were evaluated for their salivary cortisol levels, blood pressure, heart rate, and pulse rate before and after their walks in the forest. The researchers found that the practice of forest bathing in these individuals resulted in lower cortisol levels, lower blood pressure, lower heart rate, and lower pulse rate. They also found decreased activation of the sympathetic nervous system and increased activation of the parasympathetic nervous system in the forest environment as compared to the urban environment [45]. In terms of psychological effects, studies have also found that shinrin-yoku has a protective effect on mental health and mood disorders. For instance, Furuyashiki et al. found that profile of mood states (POMS) significantly improved following forest bathing among patients diagnosed with depression [46]. Shinrin-yoku may impact health outcomes in the long-run as well. James et al. found that women living in areas with more greenery have a 12% reduced mortality compared to those with less greenery [47]. Besides its beneficial effects on the brain and mind, shinrin-yoku has also been shown to increase human natural killer (NK) cell activity, NK cell production, and intracellular concentrations of anti-cancer proteins. This suggests a potential role for shinrin-yoku in cancer prevention and immunotherapy [48].

Studies have shown that forest bathing can reduce stress hormones like cortisol and adrenaline, lower blood pressure, and enhance mood, which may indirectly benefit neurological health by mitigating stress-related exacerbations of symptoms [48]. Specifically, in the context of neurological rehabilitation, a study indicated that forest therapy might contribute to functional recovery and reduce stress indicators in patients recovering from acute ischemic stroke [49]. Although direct evidence linking forest bathing to improvements in chronic neurological diseases is limited, the practice’s positive effects on mood, immune function, and stress levels suggest it could be a valuable complementary therapy.

## 5. Animal-Assisted Therapy (AAT)

Animal companionship has been used as a medical therapy adjunct starting as early as the 19th century [50]. Historically, there has been a paucity of literature regarding the use of animals in therapeutic and clinical settings. However, in recent years, animal-assisted therapy (AAT) has become more widely recognized as a scientifically valid form of therapy given emerging research interest, particularly in the realm of mental health. As a result, hospitals, care centers, and other institutions across the globe have incorporated AAT into their standard programming, especially in pediatric patient care settings [51].

A review by Charry-Sánchez et al. evaluated and confirmed the positive effect of animal therapy on various neurological conditions [52]. Another cross-sectional study of 141 active and retired members of the U.S. Military demonstrated significant reductions in post-traumatic stress disorder (PTSD) symptoms following the use of service dogs [53]. AAT demonstrates remarkable promise in the treatment of dementia patients as well. A comprehensive body of research shows a marked reduction in patient agitation and caregiver stress through initiation of controlled social interaction facilitated by trained service animals. An investigation of patients with dementia who interacted with fish, dogs, and donkeys indicated greater nutritional intake, decreased anxiety, and greater autonomy, social integration, strength, and balance in these patients [54]. There is strong evidence supporting the use of hippotherapy, which is a therapeutic treatment that utilizes horse movement to enhance physical, emotional, and cognitive functions in patients with multiple sclerosis. Patients have shown reductions in spasticity and fatigue, along with improved quality of life ratings [55]. In the treatment of stroke patients and patients with SCIs, AAT using dogs has also shown promise; however, the need for larger cohort studies is indicated [56]. Patients living with chronic pain have also been reported to experience a reduction in pain and pain-related symptoms subsequent to dog visits, presenting with decreased catecholamines and increased endorphins subsequent to these visits [57]. Patients with Parkinson’s disease, multiple sclerosis, and a history of stroke have demonstrated marked improvement in mental and physical health when AAT is utilized, although the correlation between AAT use and mental health improvement appears to be more strongly supported [58]. Equine-assisted therapy in particular has also proven useful in the improvement of self-efficacy, self-esteem, and emotional regulation skills in patients with substance use disorders [59]. In college-aged individuals, implementation of AAT utilizing registered therapy dogs has also been shown to improve student wellness, particularly in terms of inducing overall decreased blood pressure, decreased heart and respiratory rates, and lower ratings of stress and anxiety following AAT [60]. There have been broader implications for AAT use in other areas of medicine as well, including rheumatology and geriatrics [61]. It is important to note that the use of live animals in AAT is crucial; in a meta-analysis conducted by Villarreal-Zegarra et al., the researchers looked at the use of both conventional AAT as well as pet-robot interventions (PRI) in the treatment and reduction of mental health variables such as symptoms of depression. However, they found there to be very little quantitative evidence to suggest that PRI is effective in improving mental health [62]. This suggests that there may be something more to AAT than simply the shape or sound of the animal; rather, there is something inherent to interaction with a live organism that makes AAT efficacious.

AAT is also a particularly attractive option for pediatric patients. Lasa et al. compiled evidence demonstrating that children with cerebral palsy who were treated with hippotherapy showed improved muscle symmetry, self-esteem, trunk stability, limb-reaching, walking speed, and balance. In a study of patients with a variety of neurodevelopmental disorders, Lasa et al. found that therapy with a diverse set of animals (including horses, dogs, rabbits, llamas, and donkeys) resulted in increased playfulness, environmental interaction, language skills, social integration, physical affection, and a reduction in cortisol levels and behavioral problems in these patients [63]. For pediatric patients with autism, AAT holds immense potential for improvement in a diverse spectrum of areas, including “social-emotional, physical, quality of life, behavioral skills, and adaptive skills”, in addition to prosocial behavior and enhanced emotional regulation [64,65]. In the pediatric ICU, the use of therapy dogs has also demonstrated the remarkable ability to reduce patient pain, feelings of fear, and anxiety, emotions that many pediatric patients tend to experience while in unfamiliar medical environments [66]. AAT is also effective in pediatric patients with Down syndrome. Kaya et al. found a positive relationship between the use of hippotherapy and balance, functional independence, and mobility in their 34-patient cohort [67]. Although the use of AAT in an inpatient pediatric oncology setting did not appear to have a significant effect on pediatric patients in a study from Chubak et al., the researchers reasoned that this was likely due to a lack of statistical power due to a small sample size (*n* = 26). Interestingly, they did find a significant relationship between the use of therapy dog visits and their secondary outcome, parental anxiety [68]. Another study investigating anxiety reduction in pediatric oncology patients produced a similar result [69]. Therefore, despite inconclusive results in pediatric cancer patients, AAT has utility in reducing parental anxiety concerning their children. This also suggests that there may be a general role for AAT in supporting caregivers of patients with chronic illness. An emerging avenue of study concerning pediatric AAT is the use of AAT for the treatment of chronic pain. Preliminary studies reviewed by Locher et al. tentatively demonstrated that the treatment of pediatric chronic pain via AAT appears to be effective; however, this relationship warrants further study and elucidation [70].

Lifetime upkeep of therapy animals is expensive; the initial training cost of a service dog alone can reach up to $38,000 [71]. However, the numerous psychological and physiotherapeutic benefits they provide make them a viable treatment option for patients with neurological disorders. To ensure accessibility, particularly for lower-income individuals who may not afford these costs, advocating for insurance coverage or subsidized funding is crucial. By integrating therapy animal costs into insurance policies, healthcare systems can help make this effective intervention more equitable and broadly available.

## 6. Game Therapy

Stroke sequelae such as depression, cerebral edema, and musculoskeletal complications can persist indefinitely after the initial event, causing significant impairment and decreased quality of life for patients. There is no consensus on the specific pharmacotherapy used to treat decreased motor function resulting from stroke [72]. However, game therapy may be a beneficial treatment avenue for this patient population. A prospective study by Putrino et al. looked at 10 stroke survivors with upper-limb hemiparesis and assessed limb function using the Fugl-Meyer Assessment of Upper Extremity Function (FMA-UE). Patients underwent game therapy following initial assessment of the severity of their disability. The game required the patients to use therapist-designated movements to navigate an airplane avatar through obstacles, using various combinations of wrist flexion and extension, forearm pronation and supination, and ulnar and radial deviation. The patients’ FMA-UE following three 30 min sessions per week for six weeks significantly increased. They also reported that they found the games to be enjoyable and user-friendly, suggesting that this particular form of game therapy has substantial benefit in terms of functional motor recovery following stroke events [73]. Other programs, including the 3D Personalized In-home eXErgames for Rehabilitation (PIXER) system, have utilized modern technology to record the user’s appearance, generate a live model, and integrate into an “exergame”. PIXER can be used for home exercise programs (HEPs). In a study by Desai et al., 10 patients with a history of previous stroke engaged in HEPs with PIXER for one month and subsequently without PIXER for two more months. Items such as in-game performance data, measures of physical functioning (PF) including Stroke Impact Scale (SIS), Timed Up & Go (TUG) and Goal Attainment (GA) Scale obtained at baseline, 1- and 3-months were evaluated such that the researchers found that HEPs performed using PIXER significantly improved motor function following stroke [74]. Particularly in patients with upper limb function impacted by stroke, a form of “gamified rehabilitation” called ArmAble was shown to improve upper limb motor function following the administration of game therapy [75]. Some studies did not find as significant an effect; for example, a larger randomized controlled trial involving 52 stroke survivors with upper-limb hemiplegia investigated the efficacy of custom-designed hand therapy video games (HTVG) combined with contralaterally controlled functional electrical stimulation (CCFES) therapy. The researchers assessed factors such as hand dexterity, cognitive function, upper limb impairment, and activity limitation prior to and following 12 weeks of CCFES or CCFES + HTVG. At the conclusion of the study, it was found that there was no additional benefit to supplementing CCFES with HTVG therapy, suggesting that this form of game therapy may not be as effective as other forms of game therapy or may warrant further exploration [76].

Gaming innovations have also been used to improve neurological and motor function in other neurological illnesses/injuries. For instance, the Xbox gaming system has developed an adaptive controller with oversized buttons that can be pressed with appendages other than fingers. Microsoft and the Department of Veterans Affairs have partnered to test the efficacy of this controller in rehabilitating the hand–eye coordination and muscle activation of soldiers who sustained traumatic brain injuries (TBIs) and spinal cord injuries (SCIs) [77]. Computer-based cognitive interventions (CCIs) have also been shown to reduce mild cognitive decline, one of the hallmark signs of dementia. This suggests that CCI can be utilized to prevent the progression of mild cognitive impairment (MCI) to more serious neurological deficits [78]. Additionally, home-based exergaming can be used in patients with Parkinson’s disease. In a randomized controlled trial looking at improvement in gait/balance disorders in 50 Parkinson’s disease patients, Nuic et al. found that home-based exergaming in this patient population substantially reduced the clinical severity of the movement disorder. These findings suggest that at-home exergaming may hold immense potential for treating patients with Parkinson’s disease and other movement disorders [79]. Upper extremity performance, cognition, functional mobility, and trunk mobility have also been shown to improve with the use of exergames in Parkinson’s patients [80]. Constraint-Induced Movement therapy (CI therapy) has also been shown to reduce disability, increase use of the affected limb, and contribute to neural plasticity in patients with upper extremity hemiparesis post-stroke [81]. Serious game therapy (SGT) may be positively indicated in multiple sclerosis patients as well [82].

In pediatric patient populations, game therapy presents a particularly salient avenue for the management of neurological disorders. Commercial consoles and other gaming technology including the Nintendo Wii and Microsoft Xbox Kinect have been shown to improve pediatric cerebral palsy patient outcomes. Luna-Oliva et al. looked at 11 children with cerebral palsy and the use of “non-immersive virtual reality technology” to supplement conventional therapy. Following eight weeks of game rehabilitation therapy in addition to standard physiotherapy treatment, motor/process skills, balance, gait speed, running and jumping and fine/manual finger dexterity all demonstrated statistically significant improvement. This suggests that game therapy used to supplement conventional physiotherapy is clinically effective in treating pediatric patients with cerebral palsy [83]. Game therapy may also be used for pediatric early onset degenerative ataxias and other neurological gait disorders. A study featuring 10 children with progressive spinocerebellar ataxia utilized three Microsoft Xbox Kinect video games to address body coordination and balance issues observed in these patients. Following a six-week therapeutic period, researchers found a statistically significant reduction in ataxia symptoms, improved balance, and gait [84]. These findings imply that game therapy, whether used alone or in conjunction with other forms of conventional physical therapy, can result in notably improved patient outcomes and motor function following neurological injury or disorder.

Some limitations of game therapy include variable efficacy depending on the patient and the therapist skill, lengthy treatment process, and potential for distraction, especially in electronic games [85]. Therefore, game therapy should be tailored and considered on a patient-to-patient basis. However, there is abundant evidence to support the numerous benefits of game therapy, especially in treating neurological conditions.

## 7. Essential Oil/Aromatherapy

**Figure 2:**
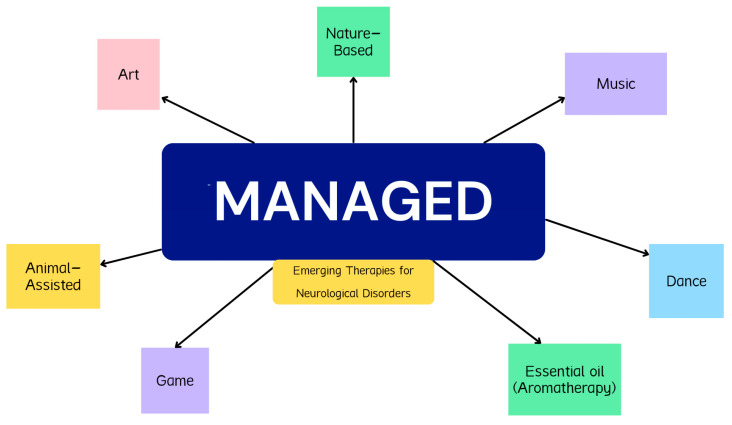
“MANAGED” Care: a mnemonic that represents the emerging therapies for neurological disorders.

While anecdotal evidence has previously implicated the sense of smell as a therapeutic avenue for nervous system disorders, recent studies have provided some empirical evidence of a place for aromatherapy in neurological treatment. Researchers have begun to elucidate the mechanism behind aromatherapy. In a study conducted by Lv et al., they found that the use of lavender, lemon, and bergamot essential oils may aid in alleviating anxiety and other mood disorders. In general, they have postulated that inhalation of essential oils transduces the smell signal to the olfactory nerve, stimulating neurons in the brain to fire neurotransmitters such as dopamine and serotonin, both of which are responsible for positively regulating mood. Based on this understanding, aromatherapy could be utilized as a potential treatment for mood and personality disorders. Chemical mediators such as monoamines, neurotrophins, cAMP, cation channels, and neuroimmune interactions have been hypothesized to convert the olfactory signal to neuromodulation of mood and mind [86]. The mechanism of neuromodulation of various body systems via the inhalation of essential oils is depicted graphically in Figure 2. When inhaled, volatile compounds from essential oils bind to olfactory receptors in the nasal cavity. These receptors transmit signals directly to the limbic system, a brain region integral to emotion and memory processing. This direct connection explains how certain scents can swiftly influence mood and induce relaxation. Some essential oil molecules may traverse the olfactory nerve pathways, reaching brain areas associated with emotional regulation. Additionally, these compounds can enter the bloodstream via alveolar absorption in the lungs, cross the blood–brain barrier, and interact with central nervous system structures [87]. Essential oils can also influence neurotransmitter systems. For instance, lavender oil has been shown to activate the GABAergic system, enhancing inhibitory neurotransmission and producing calming effects. Other oils may modulate monoamine levels, such as serotonin and dopamine, contributing to mood stabilization [88].

**Figure 3:**
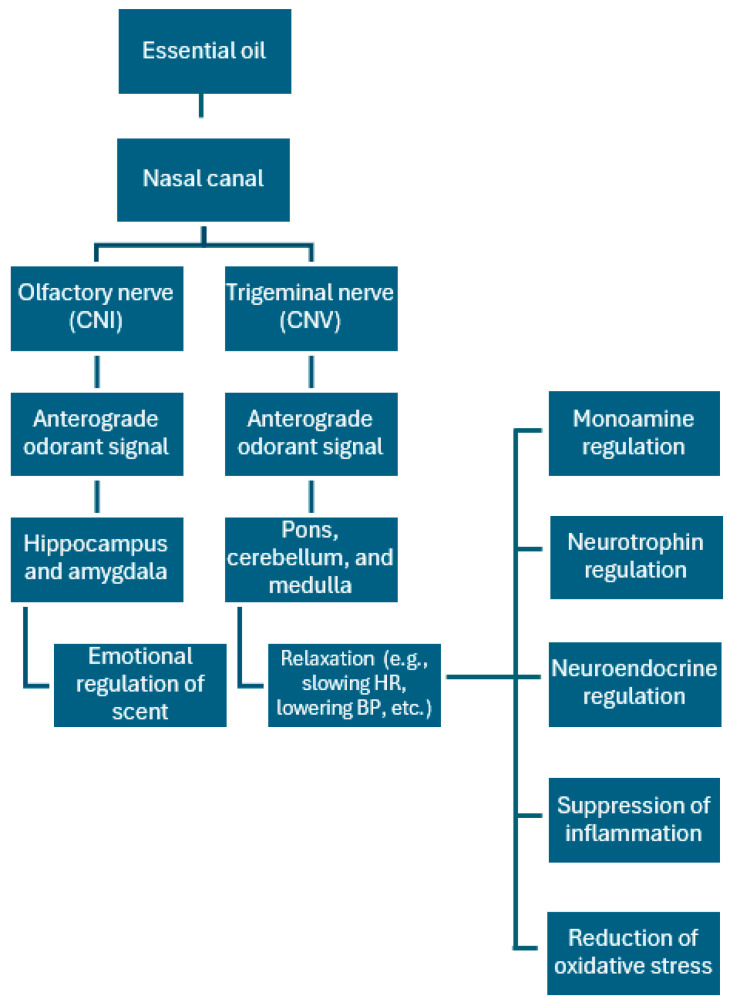
Diagram outlining the hypothesized mechanism of action of essential oils/aromatherapy on the brain causing subsequent relaxation. Essential oils pass through the nasal canal and bind to olfactory receptors. After these receptors are endocytosed, these oils are converted into chemical odorant signals via the olfactory (CNI) and trigeminal (CNV) nerves that travel to the olfactory cortex/hippocampus/amygdala and the pons/cerebellum/medulla, respectively. Hippocampus/amygdala activation activates the olfactory component of memory and mood, while activation of the pons/cerebellum/medulla trigger relaxation and depression of vital signs (e.g., decreased heart rate, blood pressure, etc.). These changes in the brain impact the neural regulation of monoamines (e.g., increasing serotonin levels), neurotrophins (e.g., increasing BDNF expression), and the neuroendocrine system (e.g., decreased cortisol levels). Essential oils have also been shown to reduce inflammation and oxidative stress in the body via this pathway.

Inhalation of certain essential oils has been associated with reduced cortisol levels, the body’s primary stress hormone, and increased parasympathetic nervous system activity, which promotes relaxation and recovery. Emerging research suggests that essential oils may promote neurogenesis and the expression of neurotrophic factors, supporting brain health and potentially contributing to their therapeutic effects [89]. Additionally, essential oils may be inhaled, ingested, or applied to the skin, allowing for multiple methods of administration. This is advantageous because it eliminates the need for intravenous or more invasive methods often required for other therapeutics. Figure 3 illustrates the proposed mechanism by which essential oils, delivered through the nasal canal, activate olfactory (CNI) and trigeminal (CNV) pathways to modulate brain regions involved in emotional regulation and relaxation. This process influences monoamine, neurotrophin, and neuroendocrine signaling, while also contributing to reduced inflammation and oxidative stress.

Qneibi et al. state that essential oils and their constituents demonstrate nervous system specificity [90]. In terms of specific scents, it appears as though several herbal and florally derived scents can induce this healing effect in neurological disorders. A review including multiple clinical studies from Zdrojewicz et al. demonstrates that the use of essential oil scents, specifically salvia, can be used to treat neurodegenerative and cognitive disorders such as Alzheimer’s disease [91]. In a randomized controlled trial, salvia was demonstrated to have significant benefit in terms of cognitive improvement in patients with Alzheimer’s disease [92]. Salvia, more widely-known by its colloquial name sage, along with coriander, may have anti-inflammatory and antioxidant action [93]. Specifically, species such as Salvia, Stachys, and Carthamus plant species in essential oils have demonstrated a protective effect in neurons against oxidative stress-induced apoptosis [94]. In a systematic review by Bavarsad et al., the authors highlight lavender as a “super-scent”; they found that lavender aromatherapy may have a positive effect on epilepsy, depression, anxiety, chronic migraines, and Alzheimer’s disease patients. Lavender’s molecular structure contains linalool, a molecule that acts on gamma-aminobutyric acid (GABA) receptors. Like other plant-based aromatherapies, it also prevents oxidative stress, a key process that occurs in the pathogenesis of Alzheimer’s disease. Lavender can also reduce the occurrence of epileptic seizures via an increase in potassium current and a decrease in sodium current, ultimately increasing GABA neurotransmission. This is essential in the pathogenesis of epilepsy, which involves an imbalance in excitatory/inhibitory neurotransmission [95].

Essential oils are commonly used for their therapeutic properties in aromatherapy and natural health practices, but they also pose several potential risks and interactions that users should be aware of. One of the most common risks is skin irritation or allergic reactions. Oils like cinnamon, oregano, and clove are highly concentrated and can cause redness, burning, or dermatitis if applied directly to the skin without proper dilution. Individuals with sensitive skin or conditions such as eczema are particularly vulnerable. Some essential oils, including bergamot, lemon, and lime, are also known to cause photosensitivity, leading to severe sunburn or skin discoloration when the treated skin is exposed to sunlight [96].

Inhaling essential oils, especially in large amounts or over long periods, can irritate the respiratory system. This is particularly concerning for individuals with asthma, chronic respiratory conditions, or children. Eucalyptus and peppermint oils, for example, may trigger bronchospasms in sensitive individuals [97]. Some essential oils, like lavender and tea tree, have been suggested to exhibit hormonal effects, potentially influencing estrogen or androgen activity, which may not be suitable for individuals with hormone-sensitive conditions [98].

## 8. Dance Movement Therapy (DMT)

Dance as a therapeutic modality is uniquely accessible to all who have the physical ability to engage in this artform. In particular, the use of dance movement therapy (DMT) has demonstrated numerous benefits for patients with dementia and Parkinsonian syndromes/movement disorders. Hokkanen et al. randomly recruited 29 patients with dementia from a nursing home: 14 with Alzheimer’s disease, eight with vascular dementia, and the remainder with unspecified/idiopathic dementia. The patients were randomized into two study groups: an experimental group that received DMT, and a control group that did not. The researchers controlled for other variables such as age, medications, etc. Initially, both groups were tested on visuospatial ability (clock drawing test) and memory (word list delayed recall task). They were also graded on the instrumental activities of daily living (IADLs) subscale. Following therapeutic intervention in the experimental group, both groups were tested again. While no significant difference was found in memory testing, the DMT-treated group outperformed the control group in both visuospatial ability and IADLs grade. The control group’s performance either remained the same or deteriorated in all three tests [99]. DMT is also beneficial in terms of improving motor performance, mobility, balance, and mood in patients with Parkinsonian syndromes. Additionally, due to its ease of accessibility and positive mood associations, patients treated with DMT demonstrate high adherence to their prescribed therapeutic regimens. DMT use has also been shown to have a low frequency of adverse events and high levels of enjoyment expressed by patients [100]. DMT as a treatment for other neurodegenerative diseases, such as Alzheimer’s disease has also demonstrated a potentially positive effect. This effect includes significant increases in physical and cognitive function, functionality, psychological outcomes, and quality of life [101]. It is generally believed that the relationship between DMT and improved outcomes in this patient population is related to increased physical movement, consistent motor skill practice, and positive associations with music. DMT may also have a synergistic effect when prescribed in combination with music therapy.

DMT has also been shown to benefit patients with schizophrenia. Previous studies used in a literature review by Biondo have suggested that DMT can help strengthen the mind–body connection and increase interoception for patients with schizophrenia. Additionally, this review suggests that treatment models that combine DMT and biofeedback may demonstrate an even greater therapeutic effect than simply DMT alone [102]. Furthermore, DMT has therapeutic applications in the regulation of bone marrow density (BMD) and balance ability in patients with schizophrenia. BMD in schizophrenic patients is of particular concern in light of the fact that schizophrenic patients are relatively prone to fracture injuries [103]. In patients with Down syndrome, use of DMT as a treatment measure to improve postural stability and fall risk appear to be somewhat effective [104]. A study by Moo et al. found benefits of in-person DMT over virtual sessions. Although there are beneficial effects in the treatment of Down syndrome for both DMT modalities, the effects appear to be markedly increased when DMT is administered in person [105]. Broadly speaking, DMT has demonstrated remarkable benefits in terms of improving aerobic capacity, passive ROM, strength, and posture control in adults with neurodevelopmental disabilities. This includes patients with cerebral palsy, autism, and Down syndrome, along with other forms of intellectual disability [106]. DMT appears to have numerous benefits in pediatric populations as well. Through a retrospective chart review, Bryl et al. found that DMT implementation in pediatric oncology patients resulted in enhanced coping with being in the hospital, reduced pain, increased physical activation, and better self-regulation [107]. In children with autism, self-guided mixed reality DMT has been shown to significantly improve movement quality and target-related responses [108].

Considering the numerous benefits of DMT, clinicians are encouraged to consider it as a treatment option when evaluating patients, especially in individuals with neurological motor disorders. Enlisting the services of certified dance movement therapists can provide an excellent, cost-effective treatment option for patients with a wide range of neurological conditions.

## 9. Conclusions

Although some of the alternative therapies discussed may necessitate larger studies to fully substantiate the link between their implementation and clinical benefits, the preliminary evidence is compelling. Table 1 summarizes some of the findings from studies on the efficacy of these alternative therapies. In the contemporary healthcare environment, where pharmacotherapies are becoming increasingly cost-prohibitive with more widespread tolerance and deleterious side effects, alternative therapies present cost-effective and largely non-invasive methods to reduce the severity of many neurological diseases. If nothing else, all these therapies are associated with improvements in patient quality of life, which is just as essential to human health as other conventional aspects of health. This is particularly relevant considering the limitations of pharmacotherapy and surgical procedures in treating and curing many neurodegenerative and neurological conditions. As we gain a deeper understanding of neural plasticity and how it is affected in individual patients by these treatments, these therapies will become even more precise and efficacious in their scope. Moving forward, future research should prioritize large-scale, randomized controlled trials to better assess the long-term efficacy and mechanisms of these interventions. Moreover, clinical practice should begin to integrate validated alternative therapies into multidisciplinary care models, especially for patients with chronic or treatment-resistant neurological conditions. Establishing standardized guidelines and training for healthcare providers can further enhance the safe and effective application of these therapies. With a collaborative, evidence-based approach, alternative treatments have the potential to play a transformative role in the future of neurological care.

## Figures and Tables

**Table 1:** Alternative therapies organized by study, type of therapy tested, neurological condition of the patients, tests/scales utilized, and quantitative scores/improvement. Neurological conditions such as Parkinson’s disease, Alzheimer’s disease, dementia, anxiety, and schizophrenia are explored in terms of scales that measure motor skills, image recognition, balance, and cognitive ability. Overall, these studies suggest that alternative therapies provide moderate benefit for patients living with neurodegenerative diseases, psychiatric illnesses, post-stroke disability, and stress/anxiety.

Study	Type of Therapy	Neurological Condition	Tests/Scales	Quantitative Improvement (Experimental)	Quantitative Improvement (Control)
Putrino et al. (2017) [73]	GT	Stroke/UL rehabilitation	FMA-UL, SUS, PACES	2.8 ± 2.1 pts (FMA-UL), 72 ± 7.9 pts (SUS), 65.8 ± 10.6 pts (PACES)	N/A
Luna-Oliva et al. (2013) [83]	GT	Cerebral palsy	GMFM, AMPS	GMFM: 85.56 ± 13.62 pts (*p* = 0.001); AMPS motor: 1.15 ± 0.28 pts (*p* = 0.001)	GMFM: 93.30 ± 13.99 pts (*p* = 0.001); AMPS motor: 2.04 ± 0.61 pts (*p* = 0.001)
López-Fernández et al. (2024) [66]	AAT	Fear, pain, anxiety	Wong–Baker scale, Child Medical Fear Scale, modified Yale Preoperatory Anxiety Scale	Wong–Baker: 2 to 0; CMFS: 1 to 0; M-YPAS: 40 to 23	CMFS: 1 to 0; M-YPAS: 32 to 23 (pre vs. post intervention)
Guan et al. (2023) [103]	DMT	Schizophrenia	BMD and BBS	BMD: 0.4 ± 0.02 to 0.5 ± 0.06; BBS: 43.1 ± 5.2 to 50.4 ± 4.5	BMD: 0.4 ± 0.03 to 0.4 ± 0.4; BBS: 44.5 ± 6.3 to 44.8 ± 7.7
Hokkanen et al. (2008) [99]	DMT	Dementia	MMSE, Word List savings score, clock drawing test, Cookie Theft picture description task from Boston Diagnostic Aphasia Test	MMSE: 12.08 ± 5.53 to 14.16 ± 6.67; Clock drawing: 1.05 ± 1.27; Word list savings: no difference compared to control; Picture description test: 14.32 ± 13.62 to 19.76 ± 8.02	MMSE: no improvement; Clock drawing test: 0.30 ± 0.48; Word list savings: no difference compared to experimental; Picture description test: 13.75 ± 12.68 to 13.4 ± 10.15
Ettinger et al. (2023) [31]	ART	Parkinson’s disease	HTP-PDS	Motor control: +1.0; visual/spatial functioning: +0.5; cognition: +1.0; motivation: +1.1; emotion: +1.5; self: +0.9; interpersonal relatedness: +0.8; creativity: +1.0	N/A
Thaut et al. (2018) [19]	MT	Parkinson’s disease	BBS, TUG	BBS: 46.6 to 51.8; TUG: 12 to 12.1 s (pre vs. post intervention at 24 weeks)	BBS: 47.8 to 51.7; TUG: 11.5 to 12 s (pre vs. post intervention at 24 wks)
Wittwer et al. (2019) [26]	MT	Supranuclear palsy	ACE-III, GDS, QS	Median ACE-III: 91; median ACE III fluency: 9; median GDS: 7; median QS: 24	Median ACE-III: 89; median ACE III fluency: 10; median GDS: 7; median QS: 22
Akhondzadeh et al. (2003) [92]	AT	Alzheimer’s disease	ADAS-cog and/or CDR	ADAS-cog: F = 4.77, d.f. = 1, *p* = 0.03; CDR-SB: F = 10.84, *p* < 0.003	N/A
Park et al. (2009) [45]	SY	Stress	SCC, PR, SBP, DBP, HF (after walking)	SCC: 15.8% decrease; PR: 3.9% decrease; SBP: 1.9% decrease; DBP: 2.1% decrease; HF: 102.0% enhancement	N/A

GT = game therapy, AAT = animal-assisted therapy, DMT = dance movement therapy, ART = art therapy, MT = music therapy, AT = aromatherapy, SY = shinrin-yoku/forest bathing, GMFM = Gross Motor Function Measure, AMPS = Assessment of Motor and Process Skills, 10 MW = 10 m walk test, GSE = General Self-Efficacy scale, BMD = bone mineral density, BBS = Berg balance scale, HTP-PDS = House–Tree–Person Parkinson Disease Scale, TUG = Timed Up and Go, ACE-III = Addenbrooke’s Cognitive Examination-III, GDS = Geriatric Depression Scale, QS = quadriceps strength, ADAS-cog = cognitive subscale of the Alzheimer’s Disease Assessment Scale, CDR = Clinical Dementia Rating, SCC = salivary cortisol concentration, PR = pulse rate, SBP = systolic blood pressure, DBP = diastolic blood pressure, HF = power levels of the high-frequency component of the heart rate variability (HRV). N/A: Information for this group was either not reported or not available in the study.

## Data Availability

This study is a literature review and does not include any new data. All sources referenced in this review are publicly available and cited in the reference list.

## References

[1] Hong G.G. (1998). Acupuncture: The Historical Basis and Its US Practitioners. Lab. Med..

[2] Guo X., Ma T. (2019). Effects of Acupuncture on Neurological Disease in Clinical- and Animal-Based Research. Front. Integr. Neurosci..

[3] Rastogi S. (2014). Rehabilitative potential of Ayurveda for neurological deficits caused by traumatic spinal cord injury. J. Ayurveda Integr. Med..

[4] Nierhaus T., Pach D., Huang W., Long X., Napadow V., Roll S., Liang F., Pleger B., Villringer A., Witt C.M. (2015). Differential cerebral response to somatosensory stimulation of an acupuncture point vs. two non-acupuncture points measured with EEG and fMRI. Front. Hum. Neurosci..

[5] Bauchner H., Fontanarosa P.B. (2018). Health Care Spending in the United States Compared With 10 Other High-Income Countries: What Uwe Reinhardt Might Have Said. JAMA.

[6] Kanwar J.R., Sriramoju B., Kanwar R.K. (2012). Neurological disorders and therapeutics targeted to surmount the blood-brain barrier. Int. J. Nanomed..

[7] Christoph Diener H., Kastrup O., Brandt T., Caplan L.R., Dichgans J., Diener H.C., Kennard C. (2003). Chapter 106—Neurological and General Side Effects of Drug Therapy. Neurological Disorders.

[8] Lee D.J., Lozano A.M. (2018). The Future of Surgical Treatments for Parkinson’s Disease. J. Park. Dis..

[9] Pathan A. (2023). Limitations of Alzheimer’s Disease Medications. NeuroPharmac. J..

[10] Rajahthurai S.D., Farrukh M.J., Makmor-Bakry M., Tan H.J., Fatokun O., Mohd Saffian S., Ramatillah D.L. (2022). Use of Complementary and Alternative Medicine and Adherence to Medication Therapy Among Stroke Patients: A Meta-analysis and Systematic Review. Front. Pharmacol..

[11] Wells R.E., Baute V., Wahbeh H. (2017). Complementary and Integrative Medicine for Neurologic Conditions. Med. Clin. N. Am..

[12] Thaut M.H. (2015). Music as therapy in early history. Prog. Brain Res..

[13] T Zaatar M., Alhakim K., Enayeh M., Tamer R. (2024). The transformative power of music: Insights into neuroplasticity, health and disease. Brain Behav. Immun. Health.

[14] Sun J., Chen W. (2015). Music therapy for coma patients: Preliminary results. Eur. Rev..

[15] Chang Y.-S., Chu H., Yang C.-Y., Tsai J.-C., Chung M.-H., Liao Y.-M., Chi M., Liu M.F., Chou K.-R. (2015). The efficacy of music therapy for people with dementia: A meta-analysis of randomised controlled trials. J. Clin. Nurs..

[16] Sihvonen A.J., Särkämö T., Leo V., Tervaniemi M., Altenmüller E., Soinila S. (2017). Music-based interventions in neurological rehabilitation. Lancet Neurol..

[17] (1998). Large infarcts in the middle cerebral artery territory. Neurology.

[18] Liao H., Jiang G., Wang X. (2015). Music therapy as a non-pharmacological treatment for epilepsy. Expert Rev. Neurother..

[19] Thaut M.H., Rice R.R., Braun Janzen T., Hurt-Thaut C.P., McIntosh G.C. (2019). Rhythmic auditory stimulation for reduction of falls in Parkinson’s disease: A randomized controlled study. Clin. Rehabil..

[20] Cubo E., Leurgans S., Goetz C.G. (2004). Short-term and practice effects of metronome pacing in Parkinson’s disease patients with gait freezing while in the ‘on’ state: Randomized single blind evaluation. Parkinsonism Relat. Disord..

[21] Duncan R.P., Earhart G.M. (2012). Randomized controlled trial of community-based dancing to modify disease progression in Parkinson disease. Neurorehabil. Neural Repair.

[22] Butala A., Li K., Swaminathan A., Dunlop S., Salnikova Y., Ficek B., Portnoff B., Harper M., Vernon B., Turk B. (2022). Parkinsonics: A Randomized, Blinded, Cross-Over Trial of Group Singing for Motor and Nonmotor Symptoms in Idiopathic Parkinson Disease. Park. Dis..

[23] Machado Sotomayor M.J., Arufe-Giráldez V., Ruíz-Rico G., Navarro-Patón R. (2021). Music Therapy and Parkinson’s Disease: A Systematic Review from 2015–2020. Int. J. Environ. Res. Public Health.

[24] O’Kelly J., Bodak R. (2016). Development of the Music Therapy Assessment Tool for Advanced Huntington’s Disease: A Pilot Validation Study. J. Music Ther..

[25] Bodeck S., Lappe C., Evers S. (2015). Tic-reducing effects of music in patients with Tourette’s syndrome: Self-reported and objective analysis. J. Neurol. Sci..

[26] Wittwer J.E., Winbolt M., Morris M.E. (2019). A Home-Based, Music-Cued Movement Program Is Feasible and May Improve Gait in Progressive Supranuclear Palsy. Front. Neurol..

[27] Chatterjee D., Hegde S., Thaut M. (2021). Neural plasticity: The substratum of music-based interventions in neurorehabilitation. NeuroRehabilitation.

[28] Li K., Cui C., Zhang H., Jia L., Li R., Hu H.-Y. (2022). Exploration of combined physical activity and music for patients with Alzheimer’s disease: A systematic review. Front. Aging Neurosci..

[29] Kim S.J., Park J.-K., Yeo M.S. (2022). Dual-Task-Based Music Therapy to Improve Executive Functioning of Elderly Patients with Early Stage Alzheimer’s Disease: A Multiple Case Study. Int. J. Environ. Res. Public Health.

[30] Matziorinis A.M., Koelsch S. (2022). The promise of music therapy for Alzheimer’s disease: A review. Ann. N. Y. Acad. Sci..

[31] Ettinger T., Berberian M., Acosta I., Cucca A., Feigin A., Genovese D., Pollen T., Rieders J., Kilachand R., Gomez C. (2023). Art therapy as a comprehensive complementary treatment for Parkinson’s disease. Front. Hum. Neurosci..

[32] Deshmukh S.R., Holmes J., Cardno A., Deshmukh S.R. (2018). Art Therapy for People with Dementia.

[33] Lo T.L.T., Lee J.L.C., Ho R.T.H. (2019). Creative Arts-Based Therapies for Stroke Survivors: A Qualitative Systematic Review. Front. Psychol..

[34] Brown S.E., Shella T., Pestana-Knight E. (2018). Development and use of the art therapy seizure assessment sculpture on an inpatient epilepsy monitoring unit. Epilepsy Behav. Case Rep..

[35] Windle G., Gregory S., Howson-Griffiths T., Newman A., O’Brien D., Goulding A. (2018). Exploring the theoretical foundations of visual art programmes for people living with dementia. Dementia.

[36] Oepen R., Gruber H. (2024). Art-based interventions and art therapy to promote health of migrant populations—A systematic literature review of current research. Arts Health.

[37] Smirnoff L., Pham K. (2024). A Role for Visual Art Therapy in the Management of Migraine. Curr. Pain Headache Rep..

[38] Raudenská J., Šteinerová V., Vodičková Š., Raudenský M., Fulková M., Urits I., Viswanath O., Varrassi G., Javůrková A. (2023). Arts Therapy and Its Implications in Chronic Pain Management: A Narrative Review. Pain Ther..

[39] Aguilar B.A. (2017). The Efficacy of Art Therapy in Pediatric Oncology Patients: An Integrative Literature Review. J. Pediatr. Nurs..

[40] Campbell M., Decker K.P., Kruk K., Deaver S.P. (2016). Art Therapy and Cognitive Processing Therapy for Combat-Related PTSD: A Randomized Controlled Trial. Art Ther. J. Am. Art Ther. Assoc..

[41] Stafstrom C.E., Havlena J., Krezinski A.J. (2012). Art therapy focus groups for children and adolescents with epilepsy. Epilepsy Behav. EB.

[42] Akhan L.U., Kurtuncu M., Celik S. (2017). The Effect of Art Therapy with Clay on Hopelessness Levels Among Neurology Patients. Rehabil. Nurs. Off. J. Assoc. Rehabil. Nurses.

[43] Kanesaki H., Watanabe K., Osugi K., Ohara H., Takada K., Kinoshita M. (2023). Utility of scratch art therapy in adult epilepsy patients with difficulties in social adaptation. Epileptic. Disord..

[44] Oliva A., Iosa M., Antonucci G., De Bartolo D. (2023). Are neuroaesthetic principles applied in art therapy protocols for neurorehabilitation? A systematic mini-review. Front. Psychol..

[45] Park B.J., Tsunetsugu Y., Kasetani T., Kagawa T., Miyazaki Y. (2010). The physiological effects of Shinrin-yoku (taking in the forest atmosphere or forest bathing): Evidence from field experiments in 24 forests across Japan. Environ. Health Prev. Med..

[46] Furuyashiki A., Tabuchi K., Norikoshi K., Kobayashi T., Oriyama S. (2019). A comparative study of the physiological and psychological effects of forest bathing (Shinrin-yoku) on working age people with and without depressive tendencies. Environ. Health Prev. Med..

[47] James P., Hart J.E., Banay R.F., Laden F. (2016). Exposure to Greenness and Mortality in a Nationwide Prospective Cohort Study of Women. Environ. Health Perspect..

[48] Li Q. (2022). Effects of forest environment (Shinrin-yoku/Forest bathing) on health promotion and disease prevention—the Establishment of “Forest Medicine”. Environ. Health Prev. Med..

[49] Lee S.-H., Sohn J.-H., Sung J.H., Han S.-W., Lee M., Kim Y., Kim J.H., Jeon J.P., Lee J.J., Kim C. (2024). The impact of forest therapy on functional recovery after acute ischemic stroke. Urban For. Urban Green..

[50] Lorraine Ernst R.N. (2014). Animal-Assisted Therapy: An Exploration of Its History, Healing Benefits, and How Skilled Nursing Facilities Can Set Up Programs. Ann. Long-Term Care.

[51] Doan T., Pennewitt D., Patel R. (2023). Animal assisted therapy in pediatric mental health conditions: A review. Curr. Probl. Pediatr. Adolesc. Health Care.

[52] Charry-Sánchez J.D., Pradilla I., Talero-Gutiérrez C. (2018). Animal-assisted therapy in adults: A systematic review. Complement. Ther. Clin. Pract..

[53] O’Haire M.E., Rodriguez K.E. (2018). Preliminary efficacy of service dogs as a complementary treatment for posttraumatic stress disorder in military members and veterans. J. Consult. Clin. Psychol..

[54] Lai N.M., Chang S.M.W., Ng S.S., Tan S.L., Chaiyakunapruk N., Stanaway F. (2019). Animal-assisted therapy for dementia. Cochrane Database Syst. Rev..

[55] Muñoz-Lasa S., López de Silanes C., Atín-Arratibel M.Á., Bravo-Llatas C., Pastor-Jimeno S., Máximo-Bocanegra N. (2019). Effects of hippotherapy in multiple sclerosis: Pilot study on quality of life, spasticity, gait, pelvic floor, depression and fatigue. Med. Clin..

[56] Machová K., Procházková R., Říha M., Svobodová I. (2019). The Effect of Animal-Assisted Therapy on the State of Patients’ Health After a Stroke: A Pilot Study. Int. J. Environ. Res. Public Health.

[57] Marcus D.A. (2013). The Science Behind Animal-Assisted Therapy. Curr. Pain Headache Rep..

[58] Mittly V., Farkas-Kirov C., Zana Á., Szabó K., Ónodi-Szabó V., Purebl G. (2023). The effect of animal-assisted interventions on the course of neurological diseases: A systematic review. Syst. Rev..

[59] Souilm N. (2023). Equine-assisted therapy effectiveness in improving emotion regulation, self-efficacy, and perceived self-esteem of patients suffering from substance use disorders. BMC Complement. Med. Ther..

[60] Andriacchi M., Hopper C., Stein A., Nye R., Taylor K. (2023). Animal-Assisted Interventions on a College Campus to Improve Wellness: Adventures With the Northern Michigan University Wildpups. J. Nurs. Educ..

[61] Kocyigit B.F., Adilbekov E., Zharmenov S., Akyol A., Yessirkepov M. (2023). Evaluating the efficacy of hippotherapy: A promising intervention in rheumatology, pain medicine and geriatrics. Rheumatol. Int..

[62] Villarreal-Zegarra D., Yllescas-Panta T., Malaquias-Obregon S., Dámaso-Román A., Mayo-Puchoc N. (2024). Effectiveness of animal-assisted therapy and pet-robot interventions in reducing depressive symptoms among older adults: A systematic review and meta-analysis. Complement. Ther. Med..

[63] Muñoz Lasa S., Máximo Bocanegra N., Valero Alcaide R., Atín Arratibel M.A., Varela Donoso E., Ferriero G. (2015). Animal assisted interventions in neurorehabilitation: A review of the most recent literature. Neurol. Engl. Ed..

[64] Beavers A., Fleming A., Shahidullah J.D. (2023). Animal-assisted therapies for autism. Curr. Probl. Pediatr. Adolesc. Health Care.

[65] Kilmer M., Hong M., Randolph D., Reichel A., Huetter S., Bowden M., Kilmer C. (2024). Animal-assisted therapy in pediatric autism spectrum disorder: A case report. Nurse Pract..

[66] López-Fernández E., Palacios-Cuesta A., Rodríguez-Martínez A., Olmedilla-Jodar M., Fernández-Andrade R., Mediavilla-Fernández R., Sánchez-Díaz J.I., Máximo-Bocanegra N. (2024). Implementation feasibility of animal-assisted therapy in a pediatric intensive care unit: Effectiveness on reduction of pain, fear, and anxiety. Eur. J. Pediatr..

[67] Kaya Y., Saka S., Tuncer D. (2023). Effect of hippotherapy on balance, functional mobility, and functional independence in children with Down syndrome: Randomized controlled trial. Eur. J. Pediatr..

[68] Chubak J., Adler A., Bobb J.F., Hawkes R.J., Ziebell R.A., Pocobelli G., Ludman E.J., Zerr D.M. (2024). A Randomized Controlled Trial of Animal-assisted Activities for Pediatric Oncology Patients: Psychosocial and Microbial Outcomes. J. Pediatr. Health Care.

[69] Mahoney A.B., Akard T.F., Cowfer B.A., Dietrich M.S., Newton J.L., Gilmer M.J. (2024). Impact of Animal—Assisted Interaction on Anxiety in Children With Advanced Cancer and Their Caregivers. J. Palliat. Med..

[70] Locher C., Petignat M., Wagner C., Hediger K., Roth B., Gaab J., Koechlin H. (2023). Animal-Assisted Psychotherapy for Pediatric Chronic Pain: Case Series of an Open Pilot Study to Test Initial Feasibility and Potential Efficacy. J. Pain Res..

[71] Kelley C., Eller C. (2017). Introduction to Animal Therapy and its Related Tax Benefits. J. Couns. Psychol..

[72] Kim Y.W. (2022). Update on Stroke Rehabilitation in Motor Impairment. Brain Neurorehabil..

[73] Putrino D., Zanders H., Hamilton T., Rykman A., Lee P., Edwards D.J. (2017). Patient Engagement Is Related to Impairment Reduction During Digital Game-Based Therapy in Stroke. Games Health J..

[74] Desai K., Prabhakaran B., Ifejika N., Annaswamy T.M. (2023). Personalized 3D exergames for in-home rehabilitation after stroke: A pilot study. Disabil. Rehabil. Assist. Technol..

[75] Ali A.S., Kumaran D.S., Unni A., Sardesai S., Prabhu V., Nirmal P., Pai A.R., Guddattu V., Arumugam A. (2024). Effectiveness of an Intensive, Functional, and Gamified Rehabilitation Program on Upper Limb Function in People With Stroke (EnteRtain): A Multicenter Randomized Clinical Trial. Neurorehabil. Neural Repair.

[76] Knutson J.S., Fu M.J., Cunningham D.A., Hisel T.Z., Friedl A.S., Gunzler D.D., Plow E.B., Busch R.M., Pundik S. (2024). Contralaterally controlled functional electrical stimulation video game therapy for hand rehabilitation after stroke: A randomized controlled trial. Disabil. Rehabil..

[77] Valdes G. Microsoft is giving Xbox Adaptive Controllers, Consoles and Games to the VA [Internet] VentureBeat 2019. https://venturebeat.com/games/microsoft-is-giving-xbox-adaptive-controllers-consoles-and-games-to-the-va/.

[78] Zuschnegg J., Schoberer D., Häussl A., Herzog S.A., Russegger S., Ploder K., Fellner M., Hofmarcher-Holzhacker M.M., Roller-Wirnsberger R., Paletta L. (2023). Effectiveness of computer-based interventions for community-dwelling people with cognitive decline: A systematic review with meta-analyses. BMC Geriatr..

[79] Nuic D., van de Weijer S., Cherif S., Skrzatek A., Zeeboer E., Olivier C., Corvol J.-C., Foulon P., Pastor J.Z., Mercier G. (2024). Home-based exergaming to treat gait and balance disorders in patients with Parkinson’s disease: A phase II randomized controlled trial. Eur. J. Neurol..

[80] Çetin B., Kılınç M., Çakmaklı G.Y. (2024). The effects of exergames on upper extremity performance, trunk mobility, gait, balance, and cognition in Parkinson’s disease: A randomized controlled study. Acta Neurol. Belg..

[81] Gauthier L.V., Kane C., Borstad A., Strahl N., Uswatte G., Taub E., Morris D., Hall A., Arakelian M., Mark V. (2017). Video Game Rehabilitation for Outpatient Stroke (VIGoROUS): Protocol for a multi-center comparative effectiveness trial of in-home gamified constraint-induced movement therapy for rehabilitation of chronic upper extremity hemiparesis. BMC Neurol..

[82] Lenne B., Degraeve B., Davroux J., Norberciak L., Kwiatkowski A., Donze C. (2023). Improving cognition in people with multiple sclerosis: Study protocol for a multiarm, randomised, blinded trial of multidomain cognitive rehabilitation using a video-serious game (E-SEP cognition). BMJ Neurol. Open.

[83] Luna-Oliva L., Ortiz-Gutiérrez R.M., Cano-de la Cuerda R., Piédrola R.M., Alguacil-Diego I.M., Sánchez-Camarero C., Martínez Culebras M.D.C. (2013). Kinect Xbox 360 as a therapeutic modality for children with cerebral palsy in a school environment: A preliminary study. NeuroRehabilitation.

[84] Ilg W., Schatton C., Schicks J., Giese M.A., Schöls L., Synofzik M. (2012). Video game-based coordinative training improves ataxia in children with degenerative ataxia. Neurology.

[85] Horne-Moyer H.L., Moyer B.H., Messer D.C., Messer E.S. (2014). The use of electronic games in therapy: A review with clinical implications. Curr. Psychiatry Rep..

[86] Lv X.N., Liu Z.J., Zhang H.J., Tzeng C.M. (2013). Aromatherapy and the central nerve system (CNS): Therapeutic mechanism and its associated genes. Curr. Drug Targets.

[87] Fung T.K.H., Lau B.W.M., Ngai S.P.C., Tsang H.W.H. (2021). Therapeutic Effect and Mechanisms of Essential Oils in Mood Disorders: Interaction between the Nervous and Respiratory Systems. Int. J. Mol. Sci..

[88] Caballero-Gallardo K., Quintero-Rincón P., Olivero-Verbel J. (2025). Aromatherapy and Essential Oils: Holistic Strategies in Complementary and Alternative Medicine for Integral Wellbeing. Plants.

[89] Cui J., Li M., Wei Y., Li H., He X., Yang Q., Li Z., Duan J., Wu Z., Chen Q. (2022). Inhalation Aromatherapy via Brain-Targeted Nasal Delivery: Natural Volatiles or Essential Oils on Mood Disorders. Front. Pharmacol..

[90] Qneibi M., Bdir S., Maayeh C., Bdair M., Sandouka D., Basit D., Hallak M. (2024). A Comprehensive Review of Essential Oils and Their Pharmacological Activities in Neurological Disorders: Exploring Neuroprotective Potential. Neurochem. Res..

[91] Zdrojewicz Z., Pypno D., Bugaj B., Cabała K., Waracki M. (2015). Applications of salvia in treating cognitive disorders and Alzheimer’s disease. Postępy Fitoter..

[92] Akhondzadeh S., Noroozian M., Mohammadi M., Ohadinia S., Jamshidi A.H., Khani M. (2003). Salvia officinalis extract in the treatment of patients with mild to moderate Alzheimer’s disease: A double blind, randomized and placebo-controlled trial. J. Clin. Pharm. Ther..

[93] Raveau R., Fontaine J., Verdin A., Mistrulli L., Laruelle F., Fourmentin S., Lounès-Hadj Sahraoui A. (2021). Chemical Composition, Antioxidant and Anti-Inflammatory Activities of Clary Sage and Coriander Essential Oils Produced on Polluted and Amended Soils-Phytomanagement Approach. Molecules.

[94] Tavakkoli M., Miri R., Jassbi A.R., Erfani N., Asadollahi M., Ghasemi M., Saso L., Firuzi O. (2014). Carthamus, Salvia and Stachys species protect neuronal cells against oxidative stress-induced apoptosis. Pharm. Biol..

[95] Bavarsad N.H., Bagheri S., Kourosh-Arami M., Komaki A. (2023). Aromatherapy for the brain: Lavender’s healing effect on epilepsy, depression, anxiety, migraine, and Alzheimer’s disease: A review article. Heliyon.

[96] Tisserand R., Young R., Tisserand R., Young R. (2014). 2—Essential oil composition. Essential Oil Safety.

[97] Posadzki P., Alotaibi A., Ernst E. (2012). Adverse effects of aromatherapy: A systematic review of case reports and case series. Int. J. Risk Saf. Med..

[98] Henley D.V., Lipson N., Korach K.S., Bloch C.A. (2007). Prepubertal Gynecomastia Linked to Lavender and Tea Tree Oils. N. Engl. J. Med..

[99] Hokkanen L., Rantala L., Remes A.M., Härkönen B., Viramo P., Winblad I. (2008). Dance and movement therapeutic methods in management of dementia: A randomized, controlled study. J. Am. Geriatr. Soc..

[100] Aguiar L.P.C., da Rocha P.A., Morris M. (2016). Therapeutic Dancing for Parkinson’s Disease. Int. J. Gerontol..

[101] Ruiz-Muelle A., López-Rodríguez M.M. (2019). Dance for People with Alzheimer’s Disease: A Systematic Review. Curr. Alzheimer Res..

[102] Biondo J. (2023). Dance/Movement Therapy as a Holistic Approach to Diminish Health Discrepancies and Promote Wellness for People with Schizophrenia: A Review of the Literature.

[103] Guan H., Zhou Z., Li X., Pan Y., Zou Z., Meng X., Guan K., Zhang L., Li Z., Li X. (2023). Dance/movement therapy for improving balance ability and bone mineral density in long-term patients with schizophrenia: A randomized controlled trial. Schizophrenia.

[104] Aleksander-Szymanowicz P., Filar-Mierzwa K., Skiba A. (2025). Effect of dance movement therapy on balance in adults with Down Syndrome. A pilot study. J. Intellect. Disabil. JOID.

[105] Moo J.T., Ho R.T. (2023). Benefits and challenges of tele-dance movement psychotherapy with children with autism and their parents. Digit. Health.

[106] Ladwig J.C., Broeckelmann E.M., Sibley K.M., Ripat J., Glazebrook C.M. (2024). A synthesis of the characteristics of dance interventions engaging adults with neurodevelopmental disabilities: A scoping review. Disabil. Rehabil..

[107] Bryl K., Tortora S., Whitley J., Kim S.-D., Raghunathan N.J., Mao J.J., Chimonas S. (2023). Utilization, Delivery, and Outcomes of Dance/Movement Therapy for Pediatric Oncology Patients and their Caregivers: A Retrospective Chart Review. Curr. Oncol..

[108] Liu W., Zhang Y., Zhang B., Xiong Q., Zhao H., Li S., Liu J., Bian Y. (2024). Self-Guided DMT: Exploring a Novel Paradigm of Dance Movement Therapy in Mixed Reality for Children with ASD. IEEE Trans. Vis. Comput. Graph..

